# Recent Progress in Pathology and Pathological Anatomy

**Published:** 1878-12

**Authors:** R. H. Fitz


### RECENT PROGRESS IN PATHOLOGY AND PATHOLOGICAL ANATOMY.

By R. H. FITZ, M.D.

### PATHOLOGY.

*Passage of Migratory Cells through Inflamed Serous Membranes.*—Arnold has endeavored to ascertain through what parts of inflamed serous membranes the amœboid cells reach the surface, and what may be the condition of the substance between the superficial endothelial cells under such circumstances. He was enabled to observe that the cement between these endothelial cells became irregular in its outline after inflammation was produced, and that the migratory cells reached the surface by passing between the endothelial cells, never through them. The cement became swollen, and showed a series of dots and rings when stained with nitrate of silver; this alteration was so extensive and so uniform that it was not to be regarded as an accidental or unimportant occurrence. On the contrary, it was thought to be a direct result of the inflammatory process, and due to an increased tension of the membrane and a more abundant flow of fluid towards the surface. It could not, however, be determined whether the cellular migration was first in point of time, being followed by the punctiform changes, or whether the reverse was the case. The general impression derived from the observation of the living object was that the alteration of the cement was primary.

*Relation between Tuberculosis and Scrofula.*—The investigations of Friedlander with regard to a local tuberculosis have been made the subject of an experimental criticism by Bollinger. He objects to the inferences drawn from experiments made upon rabbits and guinea-pigs, since a variety of causes may result in the production of affections similar to tuberculosis, though not identical with human tuberculosis or phthisis. Even if a peritoneal outgrowth of nodules resembling tubercles follows the insertion of various different objects into the abdominal cavity of these animals, it is by no means to be inferred that tuberculosis of the human species and other mammals is not due to a specific agent.

The experiments of Bollinger were made upon a goat, and a classical miliary tuberculosis of the peritonæum followed the introduction of a fluid containing cheesy particles from a tuberculous bovine lung. The resulting tubercles differed in no way from the miliary tubercles of man, and their local development was independent of the course of the blood-vessels or of the larger lymphatics. Furthermore, there were no evidences of peritonitis except at that part of the peritonæum where the wound had been made. It was therefore evident that, in the inoculated variety of tuberculosis, the miliary growths may occur in healthy tissues as well as in those subject to inflammation. He considers that this experiment justifies the view that there may result from inoculation a tuberculosis thoroughly homologous with human tuberculosis.

In the same paper he gives the history of his attempts at determining experimentally the relation between scrofula and tuberculosis. A part only of the contemplated plan was carried out, and bits of a supposed scrofulous gland were inserted within the abdomen of a goat. The animal died after several weeks, and a miliary tuberculosis of the peritonæum was found. The effect of this experiment suggested to Bollinger that the individual from whom the scrofulous gland came was suffering from a tuberculous scrofula; further examination showed that solidification and fine rales were present at the top of the right lung. The inference drawn from this experiment was that certain diseases clinically regarded as scrofula are nothing else than a beginning tuberculosis, and that a different diagnosis may be made by experiment in certain initial forms of tuberculosis which pursue the course of scrofula. This experiment also confirmed the view that the agent which is present in the cheesy mass causes tubercles but not inflammation.

Other experiments have enabled him to state that certain forms of tuberculosis, induced by feeding, bear a strong morphological resemblance to scrofula of the human species, and that in many such cases the most striking change is an alteration of the lymphatic glands, like that which occurs in scrofula and tabes mesenterica. He further considers that his experiments justify him in stating that two forms of scrofula may be distinguished: the one simple and benignant; the other a specific tubercular form, which may be regarded as a beginning tuberculosis.

*Transmission of Tuberculosis through the Air.*—At the annual meeting of the society of German naturalists and physicians, Lippl gave the results of his experiments concerning the production of tuberculosis, assuming that the introduction of an infective material is the important factor. He endeavored to compel an inhalation of sputa from tuberculous patients, the minutest particles alone being used, that the irritating effect of large fragments might be avoided. Dogs were selected as the most suitable animals for experimentation, from the rarity with which they become tuberculous. This series of experiments proved negative. When the sputum was diluted with a weak solution of salt and water, however, and this mixture inhaled, a miliary tuberculosis resulted.

Tappeiner also produced an artificial tuberculosis by causing dogs to inhale a spray composed of phthisical sputa diluted with water. Other dogs were fed with similar sputa, and were found to have acquired a miliary tuberculosis. He inferred that the human subject may also become tuberculous from the inhalation of particles from phthisical sputa suspended in air. The experiments seem to favor the view of the contagious nature of pulmonary phtisis, and to indicate that it is dangerous for phthisical patients to swallow their own sputa.

At the same meeting Schweninger called attention to the importance of these observations, if the nodules were to be regarded as actual tubercles. He had examined the specimens referred to in the above communications, and was of the opinion that they represented actual tubercles, and were entirely distinct from the appearances described by Friedlander and Wolff. The hereditary character and the contagion of tuberculosis thus received a ready explanation; for the inhalation of air expired from phthisical patients and the use of milk from tuberculous mothers furnished a more satisfactory means for transmitting the tuberculous poison than the view of predisposition or constitution.

*Changes in Bone Produced by Arsenic.*—Rabbits, fowls and pigs were fed by Gies during a period of nearly four months with small but gradually increasing doses of arsenic. The several animals grew heavier and fatter, and the epiphyseal and periosteal growth of bone became increased in those animals which, during the course of the experiments, were not fully developed. Spongy bone was everywhere replaced by compact bone; the bones of the wrist and the ankle, for instance, having been found to be composed of solid bone. A dense layer of bone was formed beneath the epiphyseal cartilage, as after feeding with phosphorus, and was especially marked at the upper epiphysis of the humerus and at the lower end of the femur. Animals confined in the same pen with those experimented upon showed similar changes of the epiphyseal layer, which fact was attributed by Gies to the absorption of arsenic eliminated from the skin and lungs of their companions. Corresponding alterations were found in animals confined in a cage, beneath the perforated floor of which arsenic was spread.

When adult animals were fed with arsenic, the cortical layer of the diaphysis became considerably thickened. More or less pronounced fatty degeneration of the heart, liver, kidneys and spleen was present, although the general nutrition of the animal was most excellent. Larger doses of arsenic gave rise to the usual phenomena of arsenical poisoning, namely, inflammatory changes in the stomach, extreme hyperæmia of the intestinal mucous membrane, and a very extensive fatty degeneration of the liver, spleen, kidneys and heart.

The young born from the rabbits were cast dead, yet were remarkable for their size, and showed the beginning of the changes above described. A considerable hypertrophy of the thymus gland was also found.

*Fatty Heart.*—Leyden makes a communication to the Berlin Medical Society on this subject in connection with the report of a case. He considers that there are three forms of fatty heart: in the first the muscular fibres are degenerated; in the second there is an excessive formation of fat tissue upon the heart; in the third there is an abundant development of fat tissue upon the heart and between its muscular bundles, but occurring in cachectic individuals, whose hearts are small and atrophied, and who have but little fat tissue.

In this last form the fat is to be considered as filling gaps, but not as a cause of disease. Objection is made to the custom of including the first two forms under one head, as is usual in the modern works on diseases of the heart, although it is admitted that fatty degeneration of the heart can only be suspected during life. This suspicion may assume a greater or less degree of probability, but the existence of a fatty heart as a definite disease cannot be certainly diagnosticated.

He reports in detail a case of fatty infiltration of the heart occurring in a corpulent person, who presented during life evidences of a feeble heart. After death both ventricles were found to be dilated, and there was an aneurismal dilatation of the apex. The presence of numerous disseminated small spots, in which the muscular fibres were separated by fat, and consequently atrophied, was considered as a cause for these lesions and for the symptoms. The fatty infiltration was supposed to have disappeared in places, leaving an atrophied portion with thickened interstitial tissue. This relation between the atrophied portion and the development of fat tissue was inferred from the more abundant accumulation of the fat towards the pericardium, and the association of a fatty infiltration of the spaces betwen the muscular bundles, while the bundles themselves were atrophied.—*Boston Med. and Surg. Journal.*

